# HPV16 E6 promoting cervical cancer progression through down‐regulation of miR‐320a to increase TOP2A expression

**DOI:** 10.1002/cam4.6875

**Published:** 2024-01-11

**Authors:** Jianing Zhang, Xiaohui Yu, Yi Guo, Daqing Wang

**Affiliations:** ^1^ Department of Gynecology Dalian Women and Children's Medical Group Dalian Liaoning China; ^2^ Department of Gynecology The First Hospital of China Medical University Shenyang Liaoning China

**Keywords:** cervical cancer, HPV16 E6, invasion, migration, miR‐320a, proliferation, TOP2A

## Abstract

**Background:**

Cervical cancer (CC) has become the fourth most common cancer worldwide and it is mainly caused by the infection of human papillomavirus (HPV), especially high‐risk HPV16. Aberrant miRNA expression in CC is closely related to HPV16 infection, and the regulation of HPV16 E6 expression can affect a variety of miRNA expression. This study aims to exploring the miRNAs involved in E6 regulation in CC.

**Methods:**

Our study screened differentially expressed miRNAs in cervical cells of HPV16 infected and uninfected cervical cancer patients by analyzing the GSE81137 dataset of the gene expression omnibus database (GEO), and identified miR‐320a that plays an anti‐tumor role and is associated with good prognosis of cervical cancer. Explore the effect of HPV16 E6 on the expression of miR‐320a in cervical cancer, and verify whether HPV16 E6 regulates the downstream target gene TOP2A expression through miR‐320a, thereby affecting cervical cancer cell proliferation, apoptosis, migration, invasion, and EMT in vitro and in vivo.

**Results:**

The bioinformatic methods selected the miR‐320a, which was differentially expressed in cervical cells from HPV16‐infected patients compared to uninfected patients. We further demonstrated that miR‐320a level was regulated by HPV16 E6, which promoted the CC cell proliferation, migration, invasion, and inhibited apoptosis. In addition, we predicted the downstream target genes of miR‐320a and confirmed that TOP2A was one of its targeting proteins. Moreover, HPV16 E6 promoted the TOP2A expression in CC cells through down‐regulating miR‐320a, leading to promoting CC development.

**Conclusions:**

We confirmed that HPV16 E6 promoted the TOP2A expression through down‐regulation of miR‐320a, thus promoting CC development, and the HPV16 E6/miR‐320a/TOP2A axis may perform as a potential target for CC treatment.

## 1 INTRODUCTION

Cervical cancer is the fourth most common cancer around the world, leading to many patients' death. There were more than 600,000 new reported CC cases and 340,000 new deaths worldwide in 2020, accounting for 6.5% and 7.7% of total cancer, respectively. Therefore, CC has become a major threat to women's health.^1^ Early‐stage diagnoses and adequate treatment of CC patients perform a better prognosis; however, most CC patients are first diagnosed at a late stage due to the lack of early detection methods.^2^ While the 5‐year survival rate for CC patients without metastasis is 91.5%, the 5‐year survival rate drops significantly to only 16.5% for those with metastatic CC.^3^ The high recurrence and mortality rates of CC are related to immune evasion, chemo‐resistance, invasion, and metastasis.^4^ Therefore, it is important to investigate the pathogenesis of CC, the potential therapeutic targeting, and early diagnostic biomarkers.

HPV infection is one of the leading causes of CC.^5^ HPV is a group of small, envelope‐free, circular, double‐stranded DNA viruses in the papillomavirus family. HPV16 is the most common type of HPV.^6^, ^7^, ^8^ HPV envelope 6(E6) protein is one of the zinc‐binding proteins containing four CXXC motifs with a length of approximately 150 amino acids, which regulates tumor progression.^9^ E6 can inhibit p53 function by integrating into the host genome^10^ In addition, E6 regulates the function of the growth arrest and DNA damage‐induced protein 34 (GADD34), protein phosphatase 1 (PP1), pro‐caspase 8 and fas‐associated protein with death domain (FADD), interfering with the cell apoptosis and growth during tumor formation and progression^10^ Therefore, E6 decreases the suppressive effect of oncogenes, leading to the development and progression of CC.

MicroRNAs (miRNAs) have emerged as important molecules in carcinogenesis. The investigation of their function during cancer formation and maintenance promises new insights into human carcinogenesis, allowing for the development of new therapeutic strategies and new diagnostic methods.^11^ miRNAs bind to the 3′‐untranslated region (UTR) of mRNAs and down‐regulate the targeting gene expression at the post‐transcriptional level, leading to translational repression or mRNA degradation.^12^ miRNAs act as either oncogenes or tumor suppressors, which can influence cellular processes such as growth, differentiation, and apoptosis by targeting and regulating the expression of multiple genes in CC.^13^, ^14^ miRNAs are also involved in various epigenetic changes and DNA methylation processes in HPV infection and CC development.^15^ Several studies have shown that aberrant miRNA expression in CC is closely associated with HPV16 infection and that targeted interference with HPV16 E6 expression can affect a variety of miRNA expressions. Wang et al. found that miR‐4454 was aberrantly highly expressed in HPV16 E6‐positive CC cells and miR‐4454 promoted the CC cell metastasis and invasion through targeting alpha/beta hydrolase domain protein 2 (ABHD2)/Nudix hydrolase 21 (Nudix hydrolase 21).^16^ Zhang et al. found that miR‐504 expression was reduced in CC cells overexpressing E6, and that HPV16 E6 promoted CC cell proliferation and invasion but inhibited apoptosis by down‐regulating miR‐504 expression.^17^ Therefore, investigating the miRNAs involved in the regulation of E6 in CC is expected to screen the potential therapeutic targets and biomarkers for CC.

In this study, we explored the differentially expressed miRNAs in cervical cells from HPV16‐infected and HPV16‐uninfected CC patients in the GSE81137 dataset of the gene expression omnibus database (GEO) to investigate the effect of HPV16 E6 on miRNA expressions in CC. Our study aimed to explore the miRNAs that are implicated in the regulation of cervical carcinogenesis through HPV16 E6, with the goal of identifying downstream target genes. This investigation could offer a theoretical foundation for early detection of CC and the development of therapeutic drugs.

## 2 MATERIALS AND METHODS

### 2.1 Tissue sample collection method

A total of 30 CC patients' tissues (20 HPV16‐positive cases and 10 HPV16‐negative cases) were collected from The First Hospital of China Medical University during the period of September 2020 to March 2022. All patients had undergone surgery for CC at stage IB1‐IIA1. The clinical staging of CC was based on the International Federation of Gynecology and Obstetrics (FIGO) standards in 2018. All patients had complete clinicopathological data and had not received adjuvant treatment such as chemotherapy, endocrine therapy, and targeted therapy before surgery. All patients had signed an informed consent form. The study was approved by the ethics committee of our hospital (2021PS605K). Fresh tissues were frozen and stored in liquid nitrogen.

### 2.2 Cell culture and treatment

HPV16‐positive CC cells SiHa and HPV16‐negative CC cells C33a were purchased from Wuhan Procell Life &Technology Co. Ltd. (CL‐0210 & CL‐0045 Wuhan). Cells were cultured in MEM medium (10,370,088, Gibco™) containing 10% fetal bovine serum (10100147C, Gibco™) and 1% penicillin (15,140,148, Gibco™) in a cell culture incubator at 37°C with 5% CO_2_. SiHa cells were transfected with knockdown HPV16 E6 or overexpression TOP2A lentivirus, and C33a cells were transfected with overexpression HPV16 E6 or knockdown TOP2A lentivirus strictly according to the lentivirus instructions (Shanghai Genechem Co., LTD.). C33a cells were transfected with miR‐320a mimics, and SiHa cells were transfected with miR‐320a inhibitor according to the Lipofectamine 3000 transfection reagent (L3000150, Invitrogen™) instructions.

### 2.3 GeneChip dataset and data analysis

The high‐throughput sequencing datasets GSE81137, GSE6791, and GSE9750 were downloaded through the GEO database (https://www.ncbi.nlm.nih.gov/geo). Three datasets were all expression analyses of arrays from Homo sapiens. The GSE81137 dataset was annotated on the platform GPL16384 including three cervical cells from HPV16‐infected CC patients and three cervical cells from HPV16‐uninfected CC patients. The platform of GSE6791 dataset was GPL570, including 20 CC tissues and eight normal cervical epithelial tissues. Moreover, the platform of GSE9750 dataset was GPL96, including 33 primary CC tumor tissues and 24 normal cervical epithelial tissues. The differential gene expression in the GEO dataset was analyzed by GEO2R. The GSE81137 dataset used HPV16‐uninfected CC patients' cervical cells as the control group and HPV16‐infected CC patients' cervical cells as the experimental group. The GSE6791 and GSE9750 datasets used normal cervical epithelial tissues as the control group and CC tissues as the experimental group. The DEGs were analyzed and screened using the GEO2R online analysis tool.

MiRNA differential expression in cancer tissues and normal tissues was analyzed using OncomiR online database (http://www.oncomir.org). The Kaplan–Meier Plotter online database (https://kmplot.com/analysis/index.php) was used to analyze the relationship between miRNA expression and overall survival of cancer patients. The prediction of downstream target genes of miRNAs was performed using ENCORI online database (https://rna.sysu.edu.cn/encori/index.php). The protein interactions of the screened miRNA and target genes were predicted by STRING online database (https://cn.string‐db.org/). PPI network was constructed and visualized using Cytoscape software, and all DEGs on the PPI network were scored using different calculation methods of CytoHubba plugin. The top 10 DEGs of the highest correlation were selected. The shared DEGs with different computational methods were selected as core genes.

### 2.4 Quantitative reverse transcription polymerase chain reaction (qRT‐PCR)

Total RNA was extracted from frozen tissues or transfected cells by Trizol reagent (15596026, Invitrogen™). MiRNA was detected according to the miRNA 1st Strand cDNA Synthesis Kit (MR101‐01/02, Vazyme Biotech Co. Ltd) instructions. MiR‐320a reverse transcription primer sequence was “5’‐GTCGTATCCAGTGCAGGGTCCGAGGTATTCGCACTGGATACGACGGAAGA‐3′“. For mRNA detection, experiments were performed according to the HiScript III RT SuperMix for qPCR Reverse Transcription Kit (R312‐01/02, Vazyme Biotech Co. Ltd) instructions. Reactions were performed according to the Taq Pro Universal SYBR qPCR Master Mix qPCR kit (Q712‐02/03, Vazyme Biotech Co. Ltd) instructions. The relative expression of the detected miRNA or mRNA was calculated by the 2^−ΔΔCT^ method using U6 or GAPDH as internal reference. The primer sequences used for PCR were showed in Table S1.

### 2.5 Western blot

The whole proteins were extracted by RIPA Lysis Buffer (P0013B, Beyotime Biotechnology). Protein concentrations were measured by BCA Protein Assay Kit (P0009, Beyotime Biotechnology). Protein extracts were separated over SDS‐polyacrylamide gel (P0012AC, Beyotime Biotechnology) and transferred to PVDF membranes. Primary antibodies specific against HPV16 E6 (1:100 dilute, 251,401, Abbiotec), TOP2A (1:10000 dilute, ab52934, Abcam), and GAPDH (1:5000 dilute, ab9485, Abcam) were used to incubate membranes overnight at 4°C. The diluted HRP‐conjugated Goat Anti‐Rabbit secondary antibodies (1:10000 dilute, ab6721, Abcam) were added to membrane and incubated for 1 h at room temperature. The results were visualized using BeyoECL Plus (P0018S, Beyotime Biotechnology) and a gel imaging system, and Image J was used to calculate the gray values of the images.

### 2.6 Cell counting kit‐8 (CCK‐8) assay

CCK‐8 assay (C0037, Beyotime Biotechnology) was performed to determine cell proliferation. Cells were inoculated in 96‐well plates at a density of 2000 cells/well and incubated for 0, 24, 48, and 72. Then, the medium was discarded. The cells were incubated with the CCK‐8 assay reagent at 37°C for 1 h. The absorbance was measured at 450 nm using a microplate reader.

### 2.7 Flow cytometric analysis for cell apoptosis examination

Cells were collected in different groups. After cell counting and dilution, 5 × 10^4^ cells were centrifuged at 800 ***g*** for 5 min. The supernatant was discarded. 195 μL of Annexin V‐FITC conjugate was added for resuspension. 5 μL of Annexin V‐FITC was added and mixed gently; then, 10 μL of propidium iodide (PI) staining solution was added and mixed gently at room temperature avoiding from light. After incubation for 20 min, cells were detected on a flow cytometer.

### 2.8 Transwell assay

Cells were collected from each group and then resuspended in serum‐free medium. 5 × 10^4^ cells were added to the upper layer of Transwell chambers coated with matrix gel (for cell invasion assay) or without matrix gel (for cell migration assay). The lower layer of chambers was added to the complete medium containing 20% fetal bovine serum. In addition, the transwell system was incubated for 48 h. Then, the transwell chambers were removed, and the culture medium was discarded and washed twice with PBS. Then, cells were fixed in methanol for 30 min and stained with 0.1% crystalline violet for 20 min. The upper layer of non‐migrated cells was gently wiped off with a cotton swab and washed for three times with PBS. The results were observed under a light microscope and photographed.

### 2.9 Animal experiment

SPF grade, BALB/c nude mice, 6–8 weeks old, weighing 18–22 g, were purchased from Beijing Vital River Laboratory Animal Technology Co., Ltd. The whole animal experiment was approved by the Experimental Animal Ethics Review Committee of China Medical University (IACAC. No. CMU2021651). The cultivated cells were diluted into a cell suspension of 5 × 10^5^ cells/100 μL, injected subcutaneously into nude mice, and continued to be reared. Then, the antagomiR‐320a, agomiR‐320a, and the corresponding control (20 mg/kg) were injected intraperitoneally 1 week after tumor cell inoculation, twice a week for 3 weeks.^18^ The tumor volume was calculated by measuring the long and short diameters of the transplanted tumors in nude mice every 3 days, and the tumor weight was weighed at 4 weeks after inoculation following with euthanasia.

### 2.10 Immunohistochemistry staining

After euthanasia of mice, fresh tumor tissues were collected and fixed in 4% paraformaldehyde. Frozen sections at 5 μm thick were prepared. Sections were blocked with 2% BSA, incubated with Ki‐67 antibody overnight at 4°C, washed with PBS, and incubated with secondary antibody for 1 h at room temperature avoiding light. After being washed with PBS, sections were stained with hematoxylin for nuclei, washed with PBS, developed in DAB, sealed with neutral gum, observed under light microscope, and photographed.

### 2.11 TUNEL assay

After euthanasia, fresh tumor tissues were taken and fixed in 4% paraformaldehyde. 5 μm thick frozen sections were prepared. Then, sections were washed in PBS, permeabilized with 0.5% Triton X‐100 for 5 min, and washed in PBS. 50 μL TUNEL assay solution containing 5 μL TdT enzyme and 45 μL fluorescent labeling solution was incubated for 1 h at 37°C avoiding light, washed in PBS, and incubated in DAPI staining solution for 30 min. After being washed with PBS, dried and sealed with anti‐fluorescence quenching sealing tablets, the slices were observed under the fluorescent microscope.

### 2.12 Dual luciferase reporter gene assay

The TOP2A 3’‐UTR wild‐type luciferase reporter gene plasmid (TOP2A‐WT) containing the binding site, the TOP2A 3’‐UTR mutant luciferase reporter gene plasmid (TOP2A‐MUT) with the binding site mutation, mimics NC and miR‐320a mimics were synthesized by Genepharm Biotech Corp. The transfection into SiHa and C33a cells was strictly according to the Lipofectamine 3000 transfection kit instructions. After 24 h cultivation, cells were collected and lysed, while the cell supernatant was collected. The relative luciferase activity was calculated by chemiluminescence detection of firefly luciferase activity and renilla kidney luciferase activity (Bio‐Glo™ Luciferase Assay System, G7940, Promega). Normalized relative luciferase activity = Firefly luciferase activity/Renilla luciferase activity.

### 2.13 AGO2‐RNA binding protein immunoprecipitation assay (RIP)

SiHa and C33a cells were collected, and cell lysates were extracted. AGO2‐ RIP assays were performed strictly according to the Imprint® RNA Immunoprecipitation Kit (RIP, Sigma‐Aldrich) instructions. TOP2A mRNA and miR‐320a were precipitated in the cell lysates with AGO2 antibody, and TOP2A mRNA and miR‐320a were detected by qRT‐PCR assay.

### 2.14 Statistical analysis

The results were processed and statistically analyzed by SPSS 22.0. The measurement data conformed to normal distribution were expressed as mean ± standard deviation. All experiments were carried out in three independent replicates. Student's *t*‐test for independent samples was used for comparison between two groups. One‐way ANOVA was used for comparison between multiple groups, and Tukey post hoc test was used for comparison between two groups. *p* < 0.05 indicated that difference was statistically significant.

## 3 RESULTS

### 3.1 HPV16 E6 significantly reduced miR‐320a level in cervical cancer cells

**FIGURE 1:**
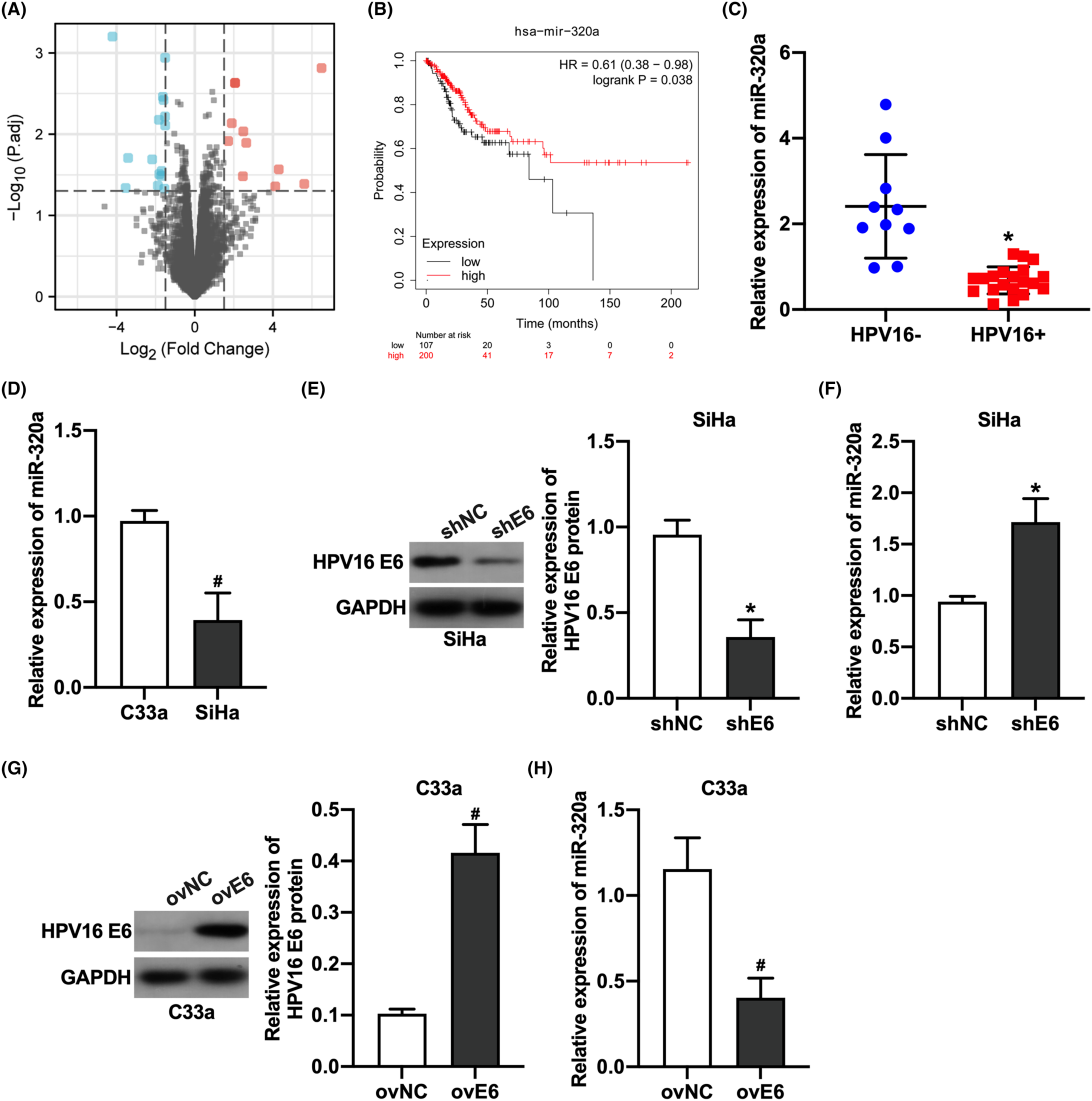
HPV16 E6 reduces the miR‐320a expression in CC cells. (A) The volcano diagram of differentially expressed miRNA in cervical tissues of HPV16‐infected and uninfected in cervical cancer patients. (B) The association between miR‐320a expression and the overall survival of CC patients. (C) qRT‐PCR results of miR‐320a expression in HPV16‐positive and negative cervical cancer tissues; *, *p* < 0.05 compared with the HPV16‐negative group. (D) qRT‐PCR results of miR‐320a expression in HPV16‐positive and negative cervical cancer cells; #, *p* < 0.05 compared with the C33a group. (E) Western blot results of HPV16 E6 expression in HPV16‐positive CC cells; *, *p* < 0.05 compared with the shNC group. (F) qRT‐PCR results of miR‐320a expression in HPV16‐positive CC cells; *, *p* < 0.05 compared with the shNC group. (G) Western blot results of HPV16 E6 expression in HPV16‐negative cervical cancer cells; #, *p* < 0.05 compared with ovNC group. (H) qRT‐PCR results of miR‐320a in HPV16‐negative CC cells; #, *p* < 0.05 compared with ovNC group.

To explore the expression profile of miRNAs in HPV16‐infected CC patients, we first analyzed the differentially expressed miRNAs in cervical tissues from HPV16‐infected CC patients and uninfected patients in the GSE81137 dataset. Twenty‐six miRNAs were differentially expressed in cervical tissues from HPV16‐infected patients, including 11 upregulated miRNAs and 15 down‐regulated miRNAs (Figure 1A). Among them, the expression of miR‐320 family members was all down‐regulated. Further analysis of the expression of miR‐320 family members in CC tissues and normal cervical tissues as well as the relationship between their expression and the overall survival rate of CC patients showed that miR‐320a was lowly expressed in CC tissues and the overall survival rate of patients with high miR‐320a expression was relatively low (Table 1, Figure 1B). To verify the effect of HPV16 infection on miR‐320a expression in CC tissues, we further examined miR‐320a expression in HPV‐negative and HPV‐positive CC tissues and cells by qRT‐PCR. The results showed that miR‐320a expression was significantly down‐regulated in HPV16‐positive CC tissues (Figure 1C) and CC cells (Figure 1D). To investigate the mechanism by which HPV16 affects miR‐320a expression in CC cells, we examined the effect of knockdown of HPV16 E6 in HPV16‐positive CC cells in SiHa cell and that of overexpression of HPV16 E6 in HPV16‐negative CC cells in C33a cell on miR‐320a expression in cells. Our results demonstrated that the knockdown of HPV16 E6 increased miR‐320a expression in SiHa cells (Figure 1E,F); in contrast, overexpression of HPV16 E6 decreased miR‐320a expression in C33a cells (Figure 1G,H). These results suggested that HPV16 E6 reduces miR‐320a expression in CC cells.

**TABLE 1:** Expression of miR‐320 family in cervical cancer tissues and normal cervical tissues.

miRNA name	Normal log2 mean expression	Tumor log2 mean expression	*p‐value*
miR‐320a	10.13	9.24	0.008
miR‐320b	2.66	2.84	0.640
miR‐320c	0.54	0.20	0.203
miR‐320d	0.54	0.39	0.668
miR‐320e	0.00	0.00	1.000

### 3.2 HPV16 E6 promotes CC cell proliferation, migration, and invasion and inhibits apoptosis through down‐regulation of miR‐320a

**FIGURE 2:**
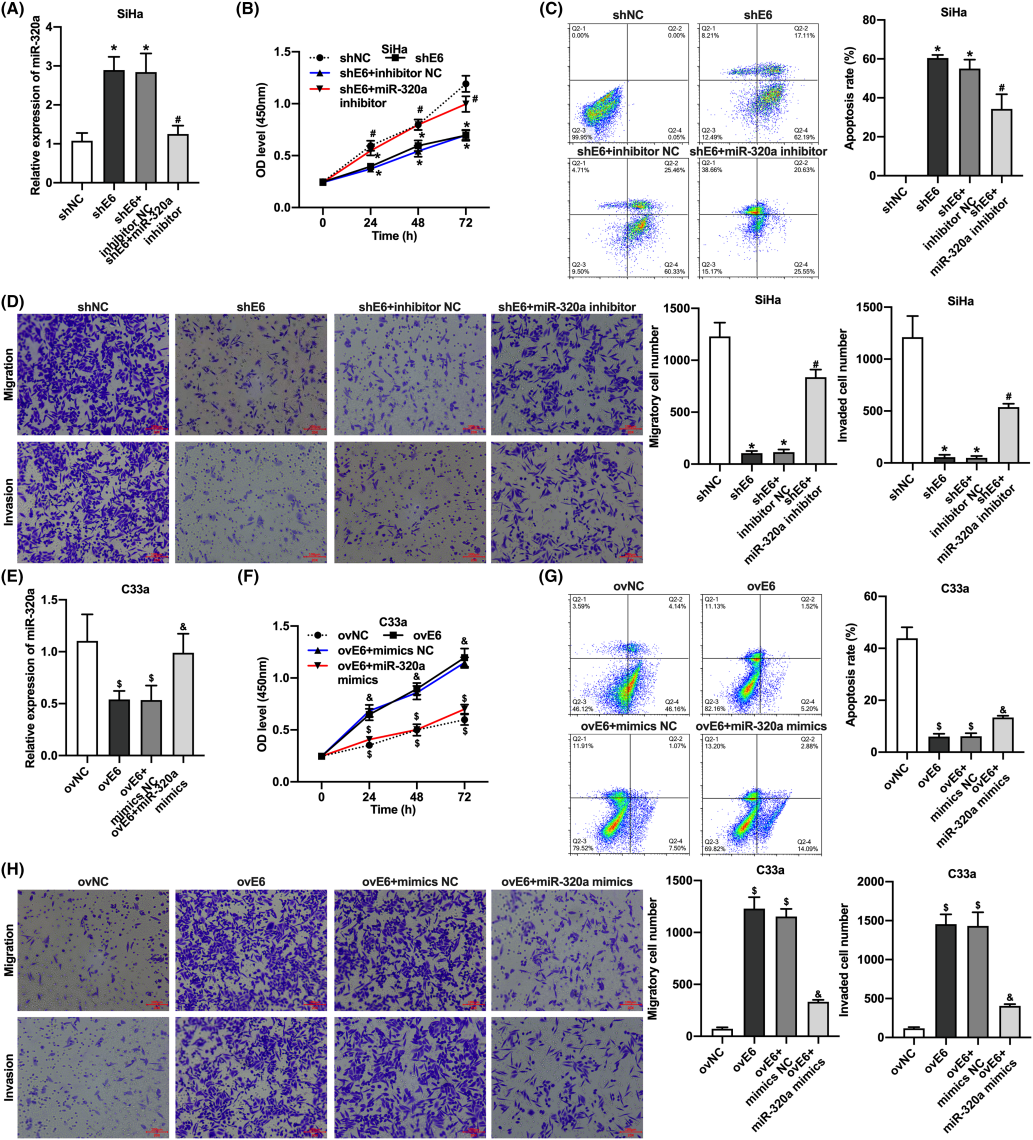
HPV16 E6 promotes proliferation, migration, and invasion and inhibits apoptosis of cervical cancer cells in vitro through down‐regulation of miR‐320a. (A) qRT‐PCR result of miR‐320a level in HPV16‐positive CC cells; (B) CCK‐8 assay of HPV16‐positive cervical cancer cells; (C) flow cytometry results for apoptosis rate of HPV16‐positive CC cells; (D) transwell assay for migration and invasion ability of HPV16‐positive cervical cancer cells; (E) qRT‐PCR results for miR‐320a level in HPV16‐negative CC cells; (F) CCK‐8 assay to detect proliferation ability of HPV16‐negative CC cells; (G) flow cytometry for detecting cell apoptosis of HPV16‐negative CC cells; (H) transwell assay to detect migration and invasion ability of HPV16‐negative CC cells. *, *p* < 0.05 compared with shNC group; #, *p* < 0.05 compared with shE6 + inhibitor NC group; $, *p* < 0.05 compared with ovNC group; &, *p* < 0.05 compared with ovE6 + mimics NC group.

To investigate whether HPV16 E6 regulates CC cell progression via miR‐320a expression, we transfected SiHa cells containing knockdown HPV16 E6 with miR‐320a inhibitor. The results showed that knockdown HPV16 E6 in SiHa cells inhibited proliferation, migration, and invasion and promoted apoptosis; however, the transfection with miR‐320a inhibitor promoted proliferation, migration, and invasion and inhibited apoptosis in SiHa cells with knockdown HPV16 E6 (Figure 2A–D). In contrast, we transfected miR‐320a mimics with C33a cells containing the overexpressing of HPV16 E6. The results demonstrated that overexpression of HPV16 E6 promoted proliferation, migration, and invasion and inhibited apoptosis in C33a cells; interestingly, the transfection of miR‐320a mimics inhibited proliferation, migration, and invasion and promoted apoptosis of C33a cells under overexpressing HPV16 E6 (Figure 2E–H). These results suggested that HPV16 E6 promotes CC cell proliferation, migration, and invasion and inhibits apoptosis through down‐regulation of miR‐320a.

### 3.3 HPV16 E6 promotes tumor growth in CC cells through down‐regulation of miR‐320a in vivo

**FIGURE 3:**
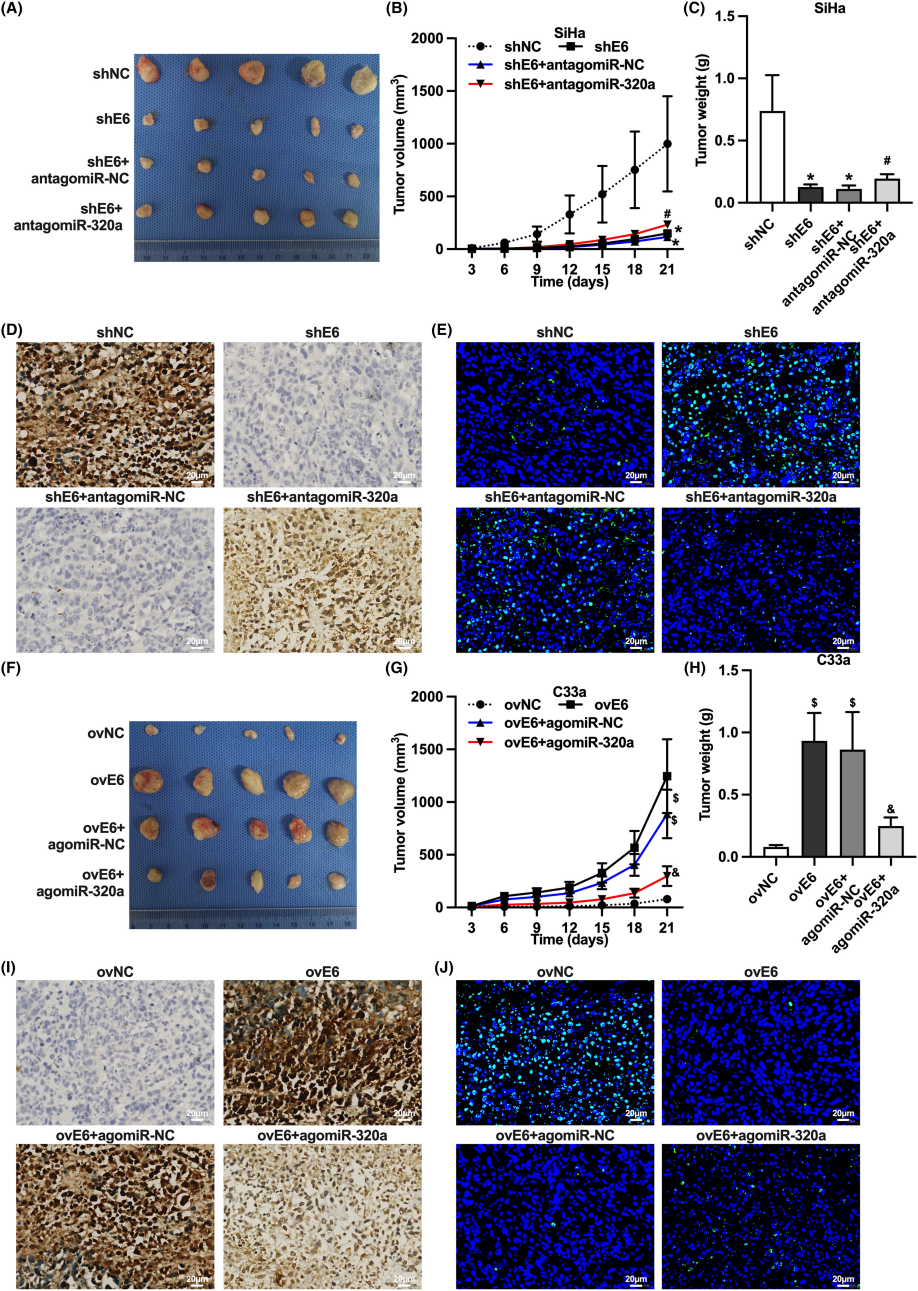
HPV16 E6 promotes tumor growth in cervical cancer cells in vivo through down‐regulation of miR‐320a. (A) Gross photograph of HPV16‐positive cervical cancer cell transplants; (B) tumor volume of HPV16‐positive cervical cancer cell transplants; (C) tumor mass of HPV16‐positive cervical cancer cell transplants; (D) Ki‐67 expression of HPV16‐positive cervical cancer cell transplants by immunohistochemistry; (E) apoptosis of HPV16‐positive cervical cancer cell transplants by TUNEL assay; (F) Gross photograph of HPV16‐negative cervical cancer cell transplant tumor; (G) tumor volume of HPV16‐negative cervical cancer cell transplant tumor; (H) tumor mass of HPV16‐negative cervical cancer cell transplant tumor; (I) immunohistochemistry detection of Ki‐67 expression of HPV16‐negative cervical cancer cell transplant tumor; (J) TUNEL assay detection of HPV16‐negative cervical cancer cell transplanted tumor cell apoptosis; *, *p* < 0.05 compared with shNC group; #, *p* < 0.05 compared with shE6 + antagomiR‐NC group; $, *p* < 0.05 compared with ovNC group; &, *p* < 0.05 compared with ovE6 + agomiR‐NC group.

We further identified whether HPV16 E6 affects tumor growth of CC cells by down‐regulating miR‐320a in vivo. We identified the effect of transfection with antagomiR‐320a on the tumor growth of transplanted SiHa cells with knockdown HPV16 E6, and found that knockdown of HPV16 E6 decreased the tumor volume and weight of transplanted SiHa cells, down‐regulated Ki‐67 expression, and increased the apoptosis rate of transplanted tumor cells. Moreover, the transfection with antagomiR‐320a increased the tumor growth of transplanted SiHa cells with knockdown HPV16 E6, upregulated transplanted tumor Ki‐67 expression, and decreased the apoptosis rate of tumor cells (Figure 3A–E). In addition, we examined the effect of transfection with agomiR‐320a on the tumor growth of transplanted C33a cells containing overexpression of HPV16 E6 and found that overexpression of HPV16 E6 increased the tumor volume and weight of transplanted C33a cells, upregulated transplanted tumor Ki‐67 expression, and decreased the apoptosis rate of transplanted tumor cells. Interestingly, the transfection of agomiR‐320a reduced the tumor volume and weight of transplanted C33a cells overexpressing HPV16 E6, down‐regulated transplanted tumor Ki‐67 expression, and increased the apoptosis rate of transplanted tumor cells (Figure 3F–J). These results confirmed that HPV16 E6 promotes tumor growth in CC cells through down‐regulation of miR‐320a in vivo.

### 3.4 miR‐320a targeting on TOP2A

**FIGURE 4:**
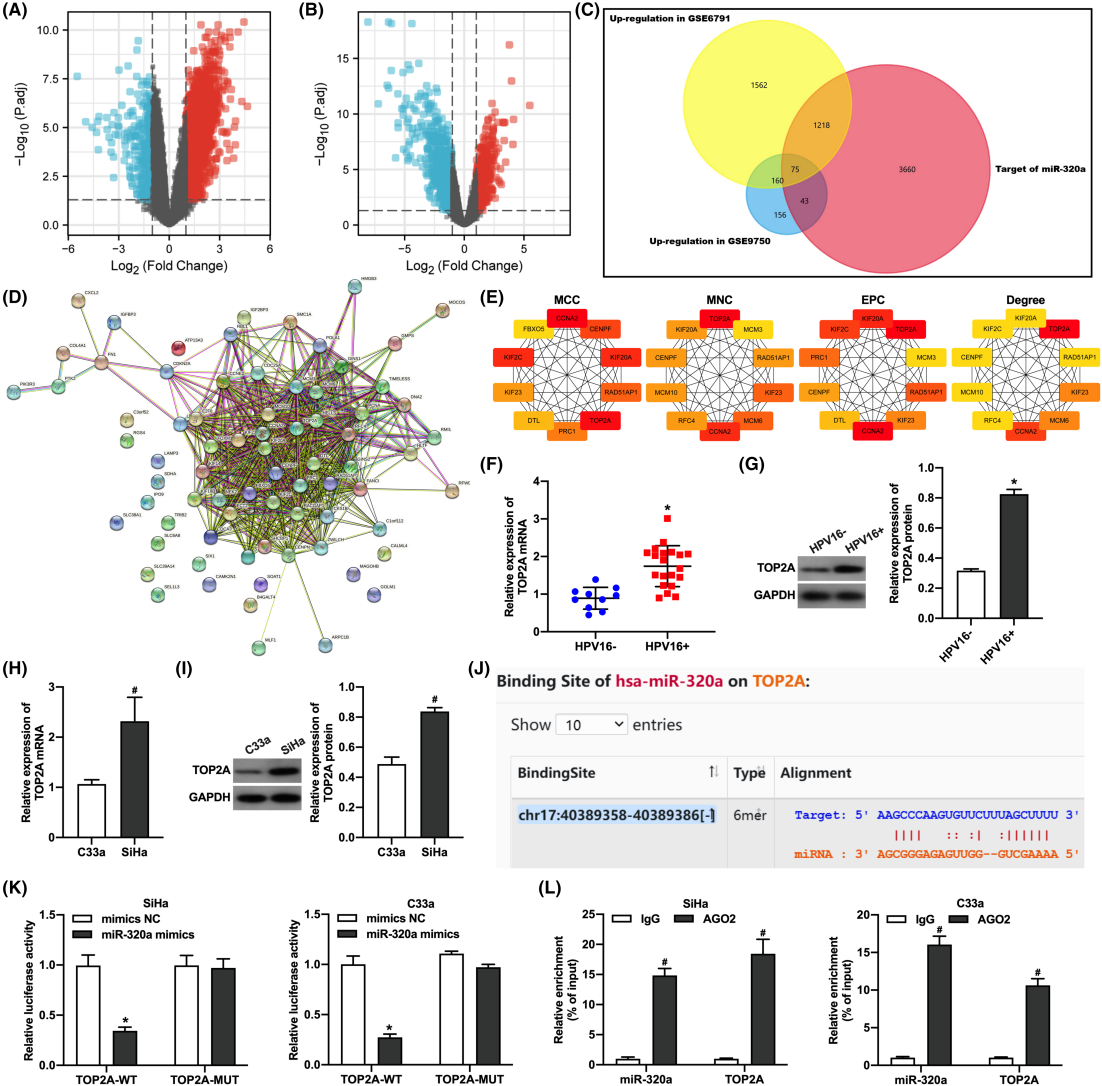
miR‐320a targeting binding to TOP2A. (A) Volcano plot of differentially expressed genes in the GSE6791 dataset. (B) Volcano plot of differentially expressed genes in the GSE9750 dataset. (C) Wayne analysis of genes upregulated in the GSE6791 and GSE9750 datasets and potential downstream target genes of miR‐320a predicted by the ENCORI database. (D) PPI map of shared genes mapped by STRING online analysis website. (E) Top 10 core genes from different computational methods. (F) qRT‐PCR detection of TOP2A mRNA expression in HPV16‐positive and negative cervical cancer tissues; *, *p* < 0.05 compared with the HPV16‐ group. (G) Western blot detection of TOP2A protein expression in HPV16‐positive and negative cervical cancer tissues; *, *p* < 0.05 compared with the HPV16‐ group. (H) qRT‐PCR to detect TOP2A mRNA expression in HPV16‐positive and negative cervical cancer cells; #, *p* < 0.05 compared with the C33a group. (I) Western blot to detect TOP2A protein expression in HPV16‐positive and negative cervical cancer cells; #, *p* < 0.05 compared with the C33a group. (J) Bioinformatics Prediction of potential binding sites of miR‐320a to TOP2A mRNA. (K) Dual luciferase reporter gene assay to validate miR‐320a binding to TOP2A mRNA; *, *p* < 0.05 compared to mimics NC group. (L) AGO2‐RIP assay to validate miR‐320a binding to TOP2A mRNA; #, *p* < 0.05 compared to IgG group.

To investigate the molecule mechanism which HPV16 E6‐regulated miR‐320a is involved in regulating CC progression, we predicted the targeting genes of miR‐320a through the ENCORI online database and analyzed those genes upregulated in CC tissues in the GSE6791 and GSE9750 datasets (Figure 4A,B). A total of 75 shared genes were screened for all three by Wayne analysis (Figure 4C). The PPI map of the shared genes (Figure 4D) was mapped by STRING online analysis website, and the core genes were screened by Cytoscape software. The results showed that the core genes screened by different computational methods included cyclin A2 (CCNA2), topoisomerase IIα (TOP2A), kinesin family member 20A (KIF20A), centromere protein F (CENPF), RAD51‐associated protein 1 (RAD51AP1), and kinesin family member 23 (KIF23) (Figure 4E, Table 2). TOP2A was selected for follow‐up in this study as it performed the highest score. We examined TOP2A expression in HPV16‐negative and positive CC tissues (Figure 4F,G) and cells (Figure 4H,I) and found that TOP2A expression was upregulated in HPV16‐positive CC tissues and CC cells. Our study also identified that miR‐320a targets binding to 3'UTR of TOP2A. Bioinformatic predictions showed the presence of a potential binding site for miR‐320a in the 3'‐UTR of TOP2A mRNA (Figure 4J). The dual luciferase reporter gene assay (Figure 4K) and AGO2‐RIP (Figure 4L) confirmed the binding of miR‐320a to the 3'‐UTR of TOP2A mRNA. The above results suggested that TOP2A is one of targeting genes of miR‐320a.

**TABLE 2:** The hub gene of the upregulated miR‐320a target gene in cervical cancer.

	MCC	MNC	Degree	EPC
1	CCNA2^a^	TOP2A^a^	TOP2A^a^	CCNA2^a^
2	TOP2A^a^	CCNA2^a^	CCNA2^a^	TOP2A^a^
3	KIF20A^a^	KIF23^a^	KIF23^a^	KIF20A^a^
3	KIF2C	MCM6	MCM6	KIF2C
5	CENPF^a^	MCM10	MCM10	RAD51AP1^a^
6	RAD51AP1^a^	KIF20A^a^	KIF20A^a^	PRC1
7	PRC1	RAD51AP1^a^	KIF2C	KIF23^a^
8	KIF23^a^	RFC4	RAD51AP1^a^	CENPF^a^
9	DTL	CENPF^a^	RFC4	DTL
10	FBXO5	MCM3	CENPF^a^	MCM3

^a^ Represents shared genes among core genes calculated by different methods.

### 3.5 HPV16 E6 promoted the TOP2A expression in CC cells through down‐regulation of miR‐320a

**FIGURE 5:**
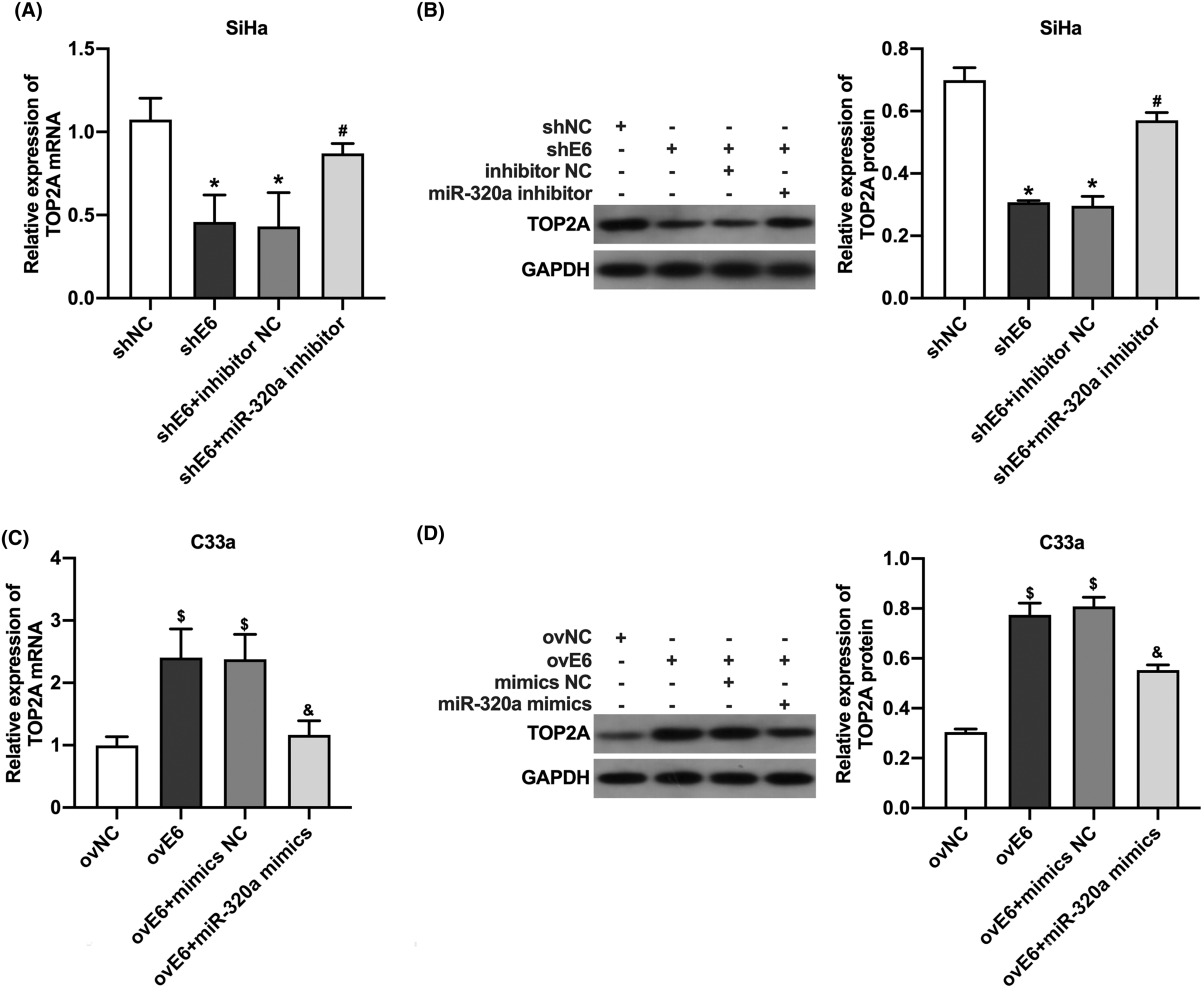
HPV16 E6 increases TOP2A expression in cervical cancer cells through down‐regulation of miR‐320a. (A) qRT‐PCR detection of TOP2A mRNA expression in HPV16‐positive cervical cancer cells; (B) western blot detection of TOP2A protein expression in HPV16‐positive cervical cancer cells; (C) qRT‐PCR detection of TOP2A mRNA expression in HPV16‐negative cervical cancer cells; (D) western blot detection of TOP2A protein expression in HPV16‐negative cervical cancer cells. *, *p* < 0.05 compared with shNC group; #, *p* < 0.05 compared with shE6 + inhibitor NC group; $, *p* < 0.05 compared with ovNC group; &, *p* < 0.05 compared with ovE6 + mimics NC group.

To confirm whether HPV16 E6 regulated TOP2A expression through down‐regulation of miR‐320a, we firstly examined the effect of transfection with miR‐320a inhibitor on TOP2A expression in SiHa cells with knockdown of HPV16 E6. The results showed that knockdown of HPV16 E6 decreased TOP2A expression in SiHa cells; In addition, the transfection with miR‐320a inhibitor could increase the TOP2A expression in SiHa cells with knockdown of HPV16 E6 (Figure 5A,B). In addition, we examined the regulatory role of transfection with miR‐320a mimics on TOP2A expression in C33a cells overexpressing HPV16 E6. The results showed that overexpression of HPV16 E6 increased TOP2A expression in C33a cells; moreover, the transfection with miR‐320a mimics decreased TOP2A expression in C33a cells overexpressing HPV16 E6 (Figure 5C,D). The above results indicated that HPV16 E6 promotes the TOP2A expression in CC cells through down‐regulation of miR‐320a.

### 3.6 HPV16 E6 promotes CC cell proliferation, migration, and invasion and inhibits apoptosis in vitro by upregulating TOP2A

**FIGURE 6:**
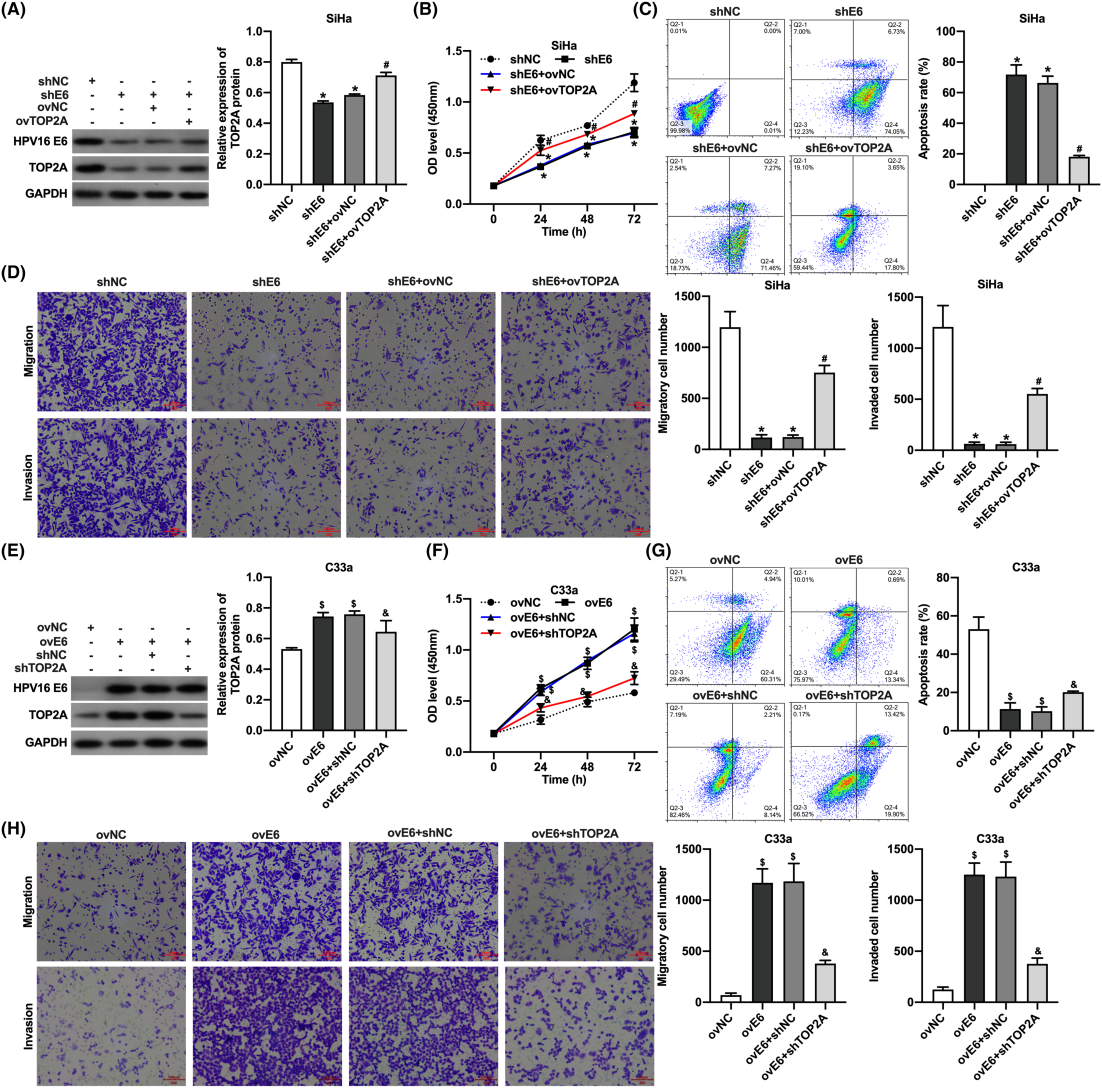
HPV16 E6 promotes proliferation, migration, and invasion and inhibits apoptosis of cervical cancer cells in vitro through upregulation of TOP2A. (A) Western blot for HPV16 E6 and TOP2A protein expression in HPV16‐positive cervical cancer cells; (B) CCK‐8 assay for proliferation of HPV16‐positive cervical cancer cells; (C) flow cytometry for apoptosis of HPV16‐positive cervical cancer cells; (D) transwell assay for migration and invasion of HPV16‐positive cervical cancer cells; (E) Western blot for HPV16 E6 and TOP2A protein expression in HPV16‐negative cervical cancer cells; (F) CCK‐8 assay for proliferation of HPV16‐negative cervical cancer cells; (G) flow cytometry for apoptosis of HPV16‐negative cervical cancer cells; (H) transwell assay for migration and invasion ability of HPV16‐negative cervical cancer cells. *, *p* < 0.05 compared with shNC group; #, *p* < 0.05 compared with shE6 + ov‐NC group; $, *p* < 0.05 compared with ovNC group; &, *p* < 0.05 compared with ovE6 + sh‐NC group.

To further verify whether HPV16 E6 promoted CC progression by regulating TOP2A expression, we transfected SiHa cells with knockdown HPV16 E6 with TOP2A overexpression lentivirus. The results demonstrated that TOP2A overexpression promoted proliferation, migration, and invasion and inhibited apoptosis in SiHa cells with knockdown HPV16 E6 (Figure 6A–D). In contrast, we transfected C33a cells overexpressing HPV16 E6 with TOP2A knockdown lentivirus. The results showed that knockdown of TOP2A inhibited the proliferation, migration, and invasion and promoted apoptosis in C33a cells overexpressing HPV16 E6 (Figure 6E–H). These results suggested that HPV16 E6 promotes CC cell proliferation, migration, and invasion and inhibits apoptosis through upregulation of TOP2A.

### 3.7 HPV16 E6 promotes tumor growth of CC cells in vivo through upregulation of TOP2A

**FIGURE 7:**
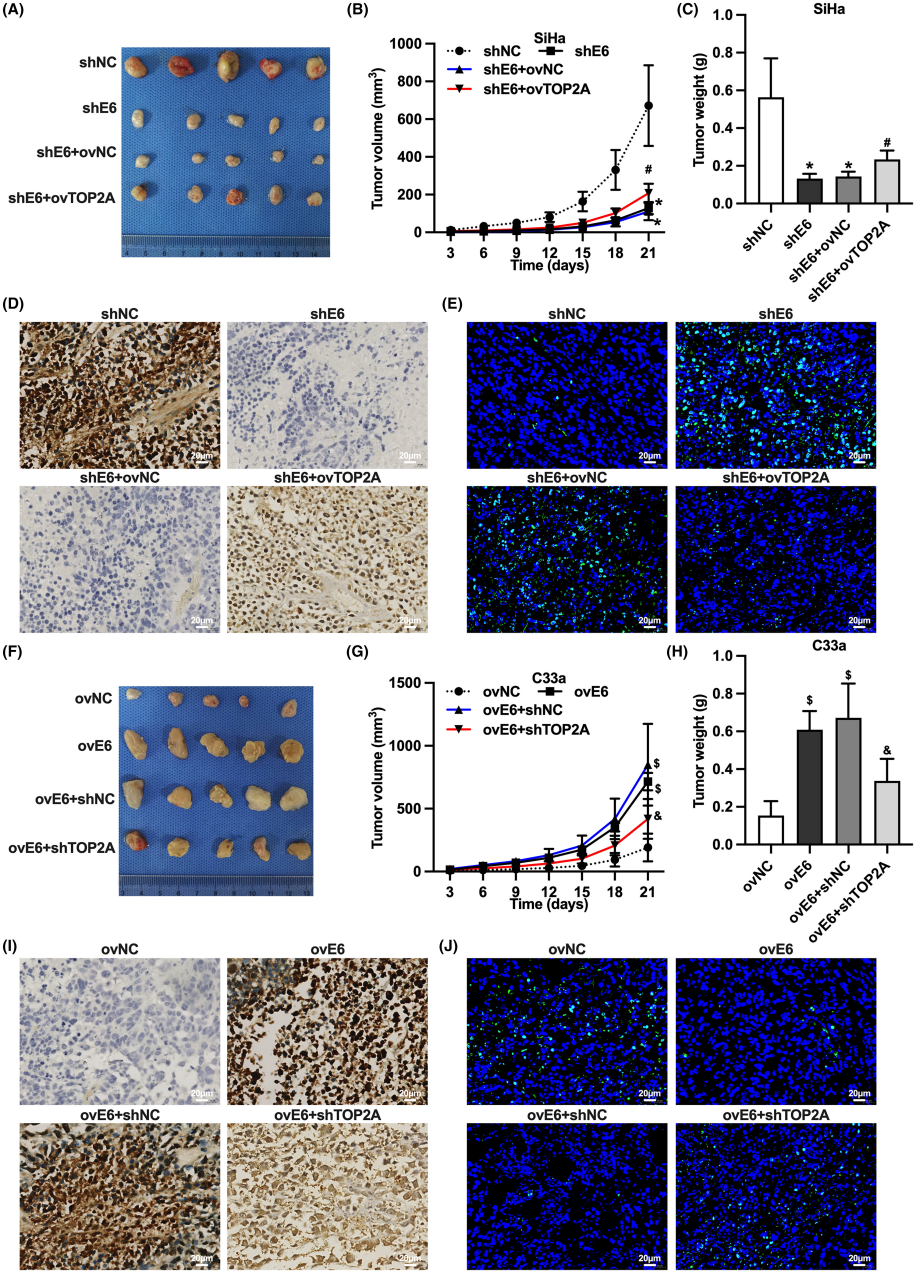
HPV16 E6 promotes in vivo tumor growth of cervical cancer cells through upregulation of TOP2A. (A) Gross photograph of HPV16‐positive cervical cancer cell transplants; (B) tumor volume of HPV16‐positive cervical cancer cell transplants; (C) tumor mass of HPV16‐positive cervical cancer cell transplants; (D) Ki‐67 expression of HPV16‐positive cervical cancer cell transplants by immunohistochemistry; (E) apoptosis of HPV16‐positive cervical cancer cell transplants by TUNEL assay; (F) gross photograph of HPV16‐negative cervical cancer cell transplants; (G) tumor volume of HPV16‐negative cervical cancer cell transplant tumor; (H) tumor mass of HPV16‐negative cervical cancer cell transplant tumor; (I) immunohistochemistry detection of Ki‐67 expression of HPV16‐negative cervical cancer cell transplant tumor; (J) TUNEL assay detection of HPV16‐negative cervical cancer cell transplanted tumor cell apoptosis; *, *p* < 0.05 compared with shNC group; #, *p* < 0.05 compared with shE6 + ov‐NC group; $, *p* < 0.05 compared with ovNC group; &, *p* < 0.05 compared with ovE6 + sh‐NC group.

We further examined whether HPV16 E6 affects tumor growth of CC cells in vivo by upregulating TOP2A. We examined the effect of TOP2A overexpression on the tumor growth of transplanted SiHa cells with knockdown HPV16 E6, and found that TOP2A overexpression increased the tumor volume and weight of SiHa cells with knockdown HPV16 E6, upregulated the Ki‐67 expression, and decreased the apoptosis rate of transplanted tumor cells (Figure 7A–E). In addition, we examined the effect of knockdown of TOP2A on the tumor growth of C33a cells overexpressing HPV16 E6. The results showed that knockdown of TOP2A reduced the tumor volume and weight of C33a cells with overexpressing HPV16 E6, down‐regulated the Ki‐67 expression, and increased the apoptosis rate of transplanted tumor cells (Figure 7F–J). These results confirmed that HPV16 E6 promotes tumor growth in CC cells through upregulation of TOP2A in vivo.

## 4 DISCUSSION

In this study, we firstly analyzed those differentially expressed miRNAs in cervical cells from patients infected with HPV16 and those from uninfected patients in the GSE81137 dataset. A total of 26 miRNAs were differentially expressed in cervical cells from patients infected with HPV16, including 11 miRNAs with upregulated expression and 15 miRNAs with down‐regulated expression. The miR‐320 family was selected as target molecules. MiR‐320 family, consisting of miR‐320a, miR‐320b, miR‐320c, miR‐320d, and miR‐320e, is closely associated with human diseases.^19^ We analyzed miRNA‐320 family expression in CC tissues and the relationship between their expression and the overall survival rate of CC patients using bioinformatics database. miR‐320a was lowly expressed in CC tissues, and the high expression of miR‐320a was related with the high overall survival rate in CC patients. Following that, our study further investigated the miR‐320a down‐regulation mediated by HPV16 E6 during CC development. Our study examined miR‐320a expression in HPV16‐positive and HPV‐negative CC tissues and cells. miR‐320a expression was down‐regulated in HPV16‐positive CC tissues and cells. In addition, overexpression of HPV16 E6 decreased miR‐320a expression in HPV16‐negative CC cells; conversely, knockdown of HPV16 E6 increased miR‐320a expression in HPV16‐positive CC cells. These results demonstrated that miR‐320a expression in CC is regulated by HPV16 E6, and miR‐320a may serve as a potential target for CC treatment and diagnosis.

Relatively few studies have reported on the role of miR‐320a in CC. Shi et al. found that miR‐320a inhibited the proliferation, migration, and invasion of CC cells by targeting on FOXM1.^20^ Similarly, Hong et al. reported that miR‐320a targeting FOXM1 inhibited the proliferation, migration, and invasion, and EMT in CC cell.^21^ It is also reported that the p38 mitogen activated protein kinases (MAPK) and c‐Jun N‐terminal kinase (JNK) pathways promoted the miR‐320a level, which further inhibited the proliferation and induced the apoptosis of Hela cell.^22^ In this study, we investigated the down‐regulation of miR‐320a induced by HPV16 E6 regulated the proliferation, apoptosis, migration, and invasion of CC cells. The overexpression of HPV16 E6 could significantly promote proliferation, migration, and invasion, but inhibit apoptosis in HPV16‐negative CC cells in vitro. In addition, the overexpression of HPV16 E6 promoted tumor growth of HPV16‐negative CC cells in tumor‐bearing mice, promoted the Ki‐67 expression, and reduced TUNEL positivity in tumor cells. Additionally, knockdown of HPV16 E6 inhibited the proliferation, migration, and invasion but promoted the apoptosis of HPV16‐positive CC cells. In vivo study, knockdown of HPV16 E6 inhibited the tumor growth of HPV16‐positive CC cells, decreased transplant tumor Ki‐67 expression, and increased the TUNEL‐positive rate of tumor cells. Transfection of miR‐320a mimics in CC cells reversed the promoting effects of overexpression of HPV16 E6 on progression in vivo and in vitro. Conversely, transfection of miR‐320a inhibitors reversed the knockdown effects of HPV16 E6 on CC cell progression in vitro and the tumor growth of HPV16‐positive CC cells in vivo. In conclusion, these results suggested that HPV16 E6 promotes CC progression through down‐regulation of miR‐320a expression.

It is well known that miRNAs inhibited gene expression by binding to the mRNAs of target genes.^23^ To investigate the molecule mechanism of miR‐320a in regulating CC progression, the downstream target genes of miR‐320a were predicted using the online bioinformatics database. The results showed that a total of 3660 potential genes were predicted. As miR‐320a has been proved to perform an anticarcinogenic role in CC, we analyzed its upregulated genes in the GSE6791 and GSE9750 datasets, and screened the upregulated miR‐320a target genes by Venn analysis. Our results identified 75 upregulated targeting genes of miR‐320a in CC. The PPI maps of 75 target genes were established by STRING, and the core genes were screened by Cytoscape software. The core genes were including CCNA2, TOP2A, KIF20A, CENPF, RAD51AP1, and KIF23, among which TOP2A performed the highest score. Therefore, TOP2A was selected to further investigate the mechanism by which HPV16 E6 promoted CC progression through down‐regulation of miR‐320a expression.

There are fewer studies about TOP2A in CC. Peres et al. reported that TOP2A was abnormally highly expressed in CC patients infected with HPV.^24^ Yang et al. reported that TOP2A was one of the important genes in CC and was associated with CC prognosis using bioinformatic analysis.^25^ Similarly, bioinformatics analysis by Zhao et al. identified TOP2A as a key candidate gene for CC progression.^26^ Wang et al. found that TOP2A was overexpressed in CC tissues compared with adjacent noncancerous tissue. In addition, the down‐regulated TOP2A expression inhibited CC cell migration and invasion, increased E‐cadherin expression, and decreased N‐cadherin expression and AKT phosphorylation. These results suggested that TOP2A overexpression promoted CC cell migration, invasion and EMT through activation of the PI3K/AKT signaling pathway.^27^ Tian et al. found that HPV58 E7 increased TOP2A expression in CC cells by upregulating E2F1 expression, thereby promoting cell proliferation and migration.^28^ However, whether TOP2A expression is regulated by the HPV16 E6 protein is not known. In the present study, we found that TOP2A was highly expressed in HPV16‐positive CC tissues and CC cells. The overexpression of HPV16 E6 increased TOP2A expression in HPV16‐negative CC cells. Conversely, the knockdown of HPV16 E6 reduced TOP2A expression in HPV16‐positive CC cells. In conclusion, the expression of TOP2A in CC was regulated by HPV16 E6. Moreover, our study demonstrated that overexpression of miR‐320a down‐regulated the TOP2A expression in HPV16‐negative CC cells with overexpressing HPV16 E6; conversely, inhibition of miR‐320a was able to upregulate TOP2A expression in HPV16‐positive CC cells with knocking down of HPV16 E6, indicating that HPV16 E6 increased TOP2A expression in HPV16‐positive CC cells by down‐regulating miR‐320a. To further confirm whether HPV16 E6 regulated the CC cell progression through TOP2A expression, we found that knockdown of TOP2A inhibited proliferation, migration, invasion, and tumor growth and promoted the apoptosis in HPV16‐negative CC cells overexpressing HPV16 E6. However, overexpression of TOP2A promoted cell proliferation, migration, invasion in vitro and tumor growth in vivo and inhibited apoptosis in HPV16‐positive CC cells with knockdown of HPV16 E6. In conclusion, we demonstrated that HPV16 E6 increased TOP2A expression in CC cells through down‐regulation of miR‐320a expression, thereby promoting CC progression.

In summary, our study proved that TOP2A expression was abnormally highly expressed in HPV16‐positive tissues and cells. miR‐320a targeted the mRNA of *TOP2A* and inhibited its expression. Moreover, HPV16 E6 increased TOP2A expression in CC cells through down‐regulation of miR‐320a expression. Therefore, the HPV16 E6‐mediated miR‐320a/TOP2A axis promotes CC progression, which may serve as a potential target for CC therapy. This study provides a theoretical basis for miR‐320a as a potential marker for diagnosing CC infected with HPV16 E6 and miR‐320a/TOP2A axis as a potential target for the treatment of cervical cancer caused by HPV infection.

## AUTHOR CONTRIBUTIONS

**Jianing Zhang:** Conceptualization (equal); writing original draft (equal). **Xiaohui Yu:** Data curation (equal); formal analysis (equal); investigation (equal). **Yi Guo:** Conceptualization (equal); funding acquisition (equal); project administration (equal); writing – review and editing (equal). **Daqing Wang:** Conceptualization (equal); funding acquisition (equal); project administration (equal); writing – review and editing (equal).

## FUNDING INFORMATION

This study was supported by the National Natural Science Foundation of China (81572054).

## CONFLICT OF INTEREST STATEMENT

The authors declare that they have no conflicts of interest.

## ETHICS APPROVAL AND CONSENT TO PARTICIPATE

The present study was approved by the Institutional Review Board and Ethics Committee of the First Hospital of China Medical University (2021PS605K). All patients signed an informed consent form. The processing of clinical tissue samples was in strict compliance with the ethical standards of the Declaration of Helsinki.

The animal experiments reported in the manuscript were approved by the Animal Experimentation Ethics Committee of China Medical University, China (CMU2021651). All experiments were performed in accordance with relevant guidelines and regulations. All methods are reported in accordance with ARRIVE guidelines (https://arriveguidelines.org) for the reporting of animal experiments.

## CONSENT FOR PUBLICATION

Not applicable.

## Supporting information


Table S1.

## Data Availability

The datasets used and/or analyzed during the current study are available from the corresponding author on reasonable request.
