# Supplementary material for: HPV16 E6 promoting cervical cancer progression through down‐regulation of miR‐320a to increase TOP2A expression
Source: Cancer Med. 2024 Jan 11;13(3):e6875. doi: 10.1002/cam4.6875 (PMC10905336; doi:10.1002/cam4.6875)
Supplement: Supplementary file 1 — Table S1. [file CAM4-13-e6875-s001.docx]

**Table S1 The** **primer** **sequences used for PCR**

| Gene name | Primer sequences (5'-3') |
| --- | --- |
| miR-320a | CGGGGAGAGGGCGAAA (forward) |
|  | AGTGCAGGGTCCGAGGTATT (reverse) |
| U6 | CTCGCTTCGGGCAGCACA (forward) |
|  | AACGCTTCACGAATTTGCGT (reverse) |
| TOP2A | TGCACCCACTTGATTGAGACAT (forward) |
|  | AGCCCTTAACCAGTACTTGCCT (reverse) |
| GAPDH | CTCCTCCACCTTTGACGC (forward) |
|  | CCACCACCCTGTTGCTGT (reverse) |
